# Pembrolizumab Failure in an Aggressive, Platinum-Resistant Primary Mediastinal Yolk Sac Tumor With Rapid Metastatic Dissemination, Spinal Cord Compression, and a Fatal Outcome

**DOI:** 10.7759/cureus.108355

**Published:** 2026-05-06

**Authors:** Salah Riyadh, Garred Cline

**Affiliations:** 1 Internal Medicine, University of Pikeville, Pikeville, USA; 2 Internal Medicine, Pikeville Medical Center, Pikeville, USA

**Keywords:** alpha-fetoprotein, extragonadal germ cell tumor, immune checkpoint inhibitor, metastatic progression, pembrolizumab resistance, platinum resistance, primary mediastinal yolk sac tumor, yolk sac tumor

## Abstract

Primary mediastinal yolk sac tumors are rare, aggressive extragonadal nonseminomatous germ cell tumors that carry a poorer prognosis than their gonadal counterparts. Outcomes are particularly dismal in platinum-resistant disease, and the role of immune checkpoint inhibitors in this setting remains poorly defined. We report the case of a 26-year-old man with a primary mediastinal yolk sac tumor who progressed despite multimodal therapy, including etoposide, ifosfamide, and cisplatin chemotherapy, high-dose carboplatin and etoposide with autologous stem cell transplantation, gemcitabine and paclitaxel, surgical debulking, and radiation therapy. Pembrolizumab was initiated for progressive disease; however, treatment was associated with rapid biochemical progression, with alpha-fetoprotein doubling from 44,053 to 90,123 ng/mL over two months, accompanied by radiographic worsening of mediastinal, chest wall, and osseous metastases. Shortly thereafter, he developed acute bilateral lower extremity weakness and urinary retention. Magnetic resonance imaging demonstrated new and enlarging multifocal spinal metastases with severe epidural extension and spinal canal stenosis, including recurrence within a prior laminectomy bed and new cervical involvement. Despite palliative radiation, he remained paraplegic and experienced continued systemic progression with central nervous system dissemination. No further disease-directed therapies were available, and he ultimately died from progressive metastatic disease. This case highlights the aggressive biology of platinum-resistant primary mediastinal yolk sac tumors, illustrates primary resistance to programmed death-1 inhibition, and underscores the urgent need for more effective salvage strategies in this high-risk population.

## Introduction

Primary mediastinal nonseminomatous germ cell tumors (PMNSGCTs) are rare extragonadal malignancies that behave more aggressively than gonadal germ cell tumors and are associated with inferior outcomes, particularly in nonseminomatous histologies such as the yolk sac tumor [1]. Reported outcomes are significantly worse in mediastinal primaries, with long-term survival rates substantially lower than those observed in gonadal germ cell tumors [1,2]. Mediastinal germ cell tumors represent a small subset of germ cell malignancies, yet they comprise an important category of anterior mediastinal tumors and frequently present with bulky disease and markedly elevated alpha-fetoprotein (AFP) levels [1,2]. Even with multimodal therapy, including cisplatin-based chemotherapy followed by surgical resection when feasible, relapse risk remains high, and prognosis declines substantially once platinum resistance develops. Platinum-resistant disease is generally defined as progression during or within a short interval after cisplatin-based therapy, in contrast to platinum-sensitive disease, which demonstrates a more durable response [1].

Beyond clinical aggressiveness, PMNSGCTs appear to be biologically distinct, with molecular features linked to treatment resistance and poor outcomes [3]. As a result, patients who progress after first-line therapy often exhaust effective systemic options quickly, and durable control becomes uncommon.

Immune checkpoint inhibitors have transformed outcomes in many solid tumors, prompting off-label or investigational use in heavily pretreated germ cell tumors. However, clinical evidence to date suggests limited activity of programmed death-1 (PD-1) pathway inhibition in platinum-resistant germ cell malignancies, with low objective response rates and frequent early progression [4,5]. Combination checkpoint strategies have also shown minimal benefit in unselected platinum-resistant germ cell tumor populations [6]. Consequently, characterizing clinical patterns of immunotherapy resistance, including rapid biochemical and radiographic progression, remains important for setting expectations, guiding monitoring strategies, and emphasizing the need for novel salvage approaches.

We report a case of aggressive platinum-resistant primary mediastinal yolk sac tumor demonstrating apparent primary resistance to pembrolizumab with fulminant metastatic progression complicated by malignant spinal cord compression and fatal outcome.

## Case presentation

**Figure 1:**
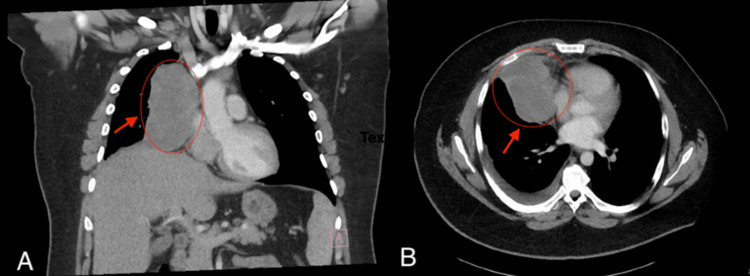
Initial contrast-enhanced CT chest demonstrating a large right anterior mediastinal mass Contrast-enhanced CT chest obtained in January 2024, demonstrating an 11.1 × 9.8 × 7.9 cm right anterior mediastinal mass with mass effect on the right heart border. (A) Coronal CT image illustrating the superior-inferior extent of the mass within the anterior mediastinum. (B) Axial CT image demonstrating displacement of the right cardiac border and adjacent mediastinal structures CT: computed tomography

A 26-year-old man with no significant prior medical history initially presented in January 2024 with right shoulder pain and progressive shortness of breath. A chest radiograph demonstrated mediastinal widening. Subsequent computed tomography (CT) of the chest obtained in January 2024 revealed a large right anterior mediastinal mass measuring 11.1 × 9.8 × 7.9 cm, contiguous with the right hilum and anterior pleura, with associated mediastinal and hilar adenopathy and mass effect on the right heart border (Figure 1). Positron emission tomography (PET) imaging demonstrated a hypermetabolic anterior mediastinal mass with metastatic adenopathy.

A CT-guided biopsy was initially nondiagnostic, necessitating a subsequent surgical biopsy for definitive diagnosis. The patient subsequently underwent right video-assisted thoracoscopic surgery with anterior thoracotomy and incisional biopsy in March 2024. Pathology demonstrated yolk sac tumor, with tumor cells positive for AFP, AE1/AE3, c-kit, and SALL4, and negative for CD30, OCT3/4, and beta-human chorionic gonadotropin, consistent with nonseminomatous germ cell tumor of mediastinal origin. Serum AFP at diagnosis measured 57,618 ng/mL.

He was treated with four cycles of etoposide, ifosfamide, and cisplatin chemotherapy, initiated in April 2024. Serum AFP declined during induction chemotherapy but remained elevated and did not normalize prior to surgical resection. In July 2024, he underwent right hemiclamshell mediastinal resection. Surgical pathology demonstrated residual primary mediastinal yolk sac tumor with extensive necrosis and <5% viable tumor; surgical margins were negative. Postoperatively, AFP decreased to 64.7 ng/mL in July 2024.

Within weeks, AFP rose again to 374 ng/mL. He subsequently received tandem high-dose carboplatin and etoposide, followed by autologous stem cell transplantation in September 2024. AFP initially decreased to 10.9 ng/mL but rose again by December 2024, with PET imaging demonstrating disease progression.

Between January 2025 and May 2025, he received gemcitabine and paclitaxel as third-line salvage chemotherapy following prior high-dose carboplatin and etoposide with autologous stem cell transplantation. AFP continued to rise, peaking above 10,000 ng/mL, with imaging demonstrating recurrent chest wall disease with rib erosion and progressive metastatic involvement.

**Figure 2:**
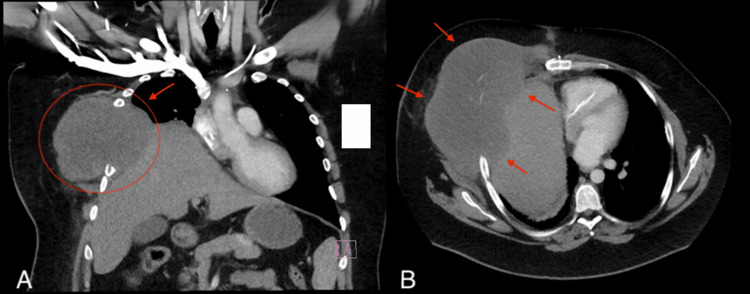
CT chest demonstrating interval progression of mediastinal disease during pembrolizumab therapy Contrast-enhanced CT chest obtained in October 2025 during pembrolizumab therapy, demonstrating marked interval enlargement of the right anterior mediastinal mass with chest wall extension and mass effect on the right heart border. (A) Coronal view illustrating increased tumor bulk. (B) Axial view demonstrating an extensive anterior mediastinal mass with displacement of adjacent mediastinal structures CT: computed tomography

Given progressive platinum-resistant disease and exhaustion of standard salvage treatment options, pembrolizumab (200 mg every three weeks) was initiated in August 2025 as an off-label therapy, as it is not currently FDA-approved for germ cell tumors. At the time of immunotherapy initiation, AFP measured 44,053 ng/mL. Despite four cycles of pembrolizumab, AFP increased rapidly to 90,123 ng/mL by October 2025. CT imaging obtained in October 2025 (Figure 2) demonstrated new mediastinal lesions and osseous metastases, with increasing pulmonary and hepatic nodules consistent with metastatic disease during PD-1 inhibition.

**Figure 3:**
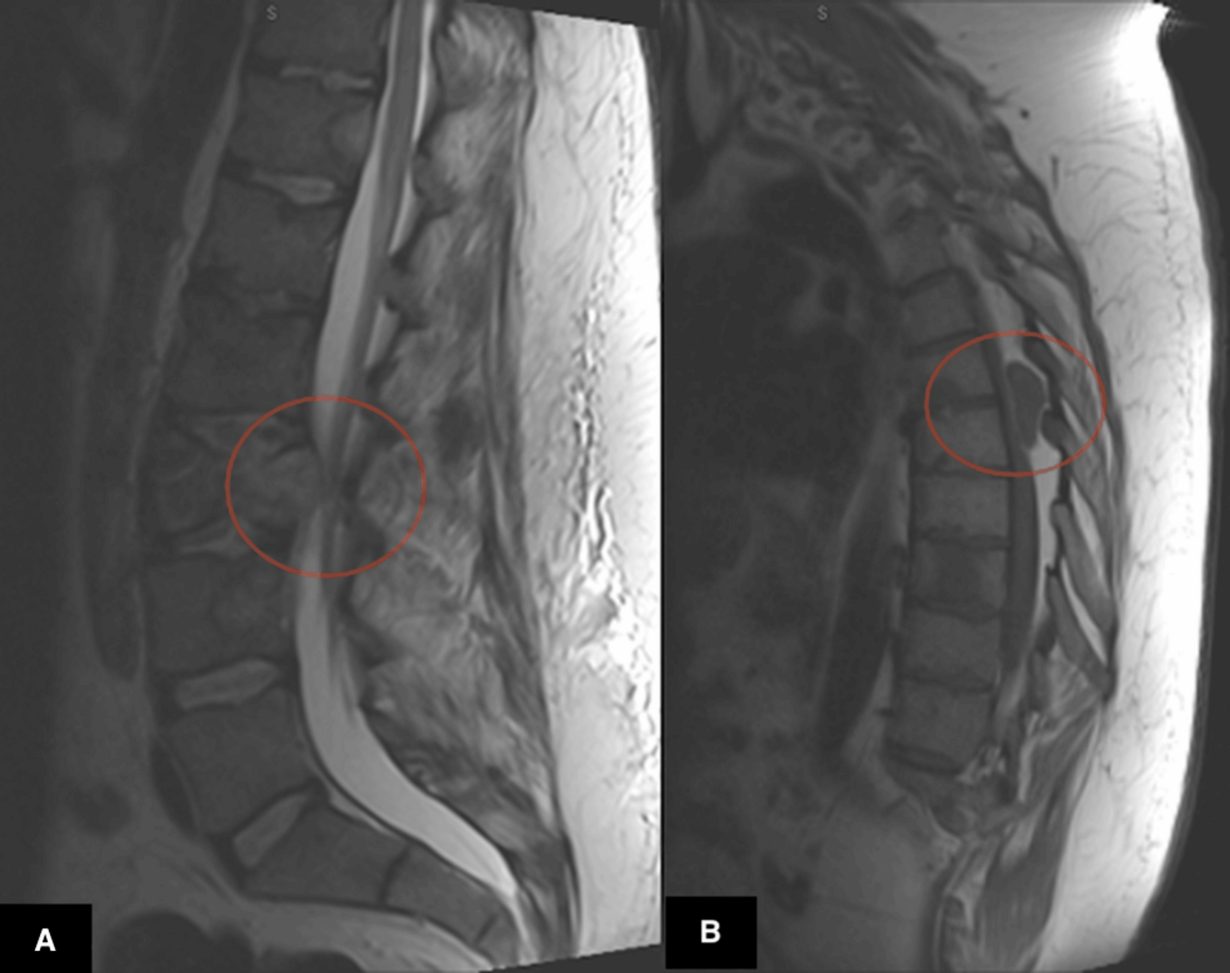
Sagittal T2-weighted MRI of the thoracic and lumbar spine obtained in November 2025 demonstrating epidural metastatic disease with spinal canal stenosis Magnetic resonance imaging of the thoracic and lumbar spine, demonstrating an epidural tumor with spinal canal compromise. (A) Sagittal lumbar spine image demonstrating a pathologic compression fracture of the L3 vertebral body with associated expansile soft tissue mass extending into the epidural space and impinging upon the thecal sac, producing moderate to severe spinal canal stenosis. (B) Sagittal thoracic spine image demonstrating an enhancing extradural mass in the posterior spinal canal at the T6-T7 level measuring approximately 3.4 × 1.1 × 1.1 cm, resulting in epidural spinal canal narrowing consistent with metastatic epidural disease MRI: magnetic resonance imaging

In November 2025, due to progressive thoracic spine involvement and concern for impending cord compression, the patient underwent T6-T7 laminectomy with tumor debulking. Magnetic resonance imaging (MRI) of the thoracic spine obtained in November 2025 (Figure 3) demonstrated numerous osseous metastases throughout the thoracic and lumbar vertebrae, an enhancing epidural mass at T6-T7 producing moderate spinal canal stenosis, expansile rib lesions, multiple pulmonary nodules, and several hepatic masses measuring up to 5.1 cm consistent with systemic metastatic disease.

**Figure 4:**
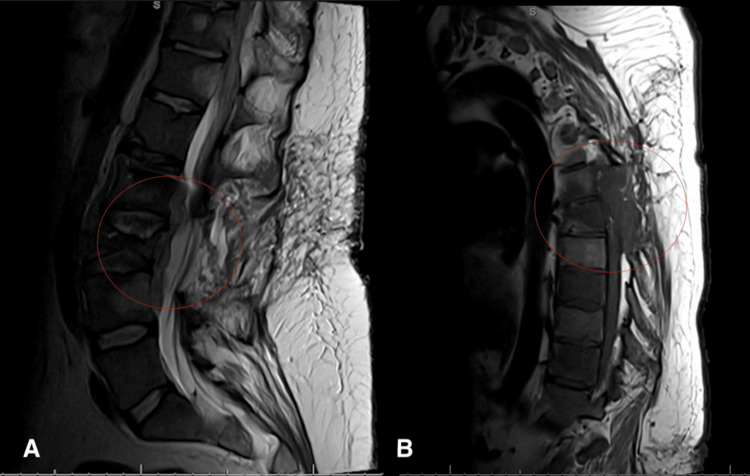
Sagittal T2-weighted magnetic resonance imaging demonstrating progressive metastatic epidural disease with multilevel spinal canal stenosis Sagittal T2-weighted MRI of the thoracic and lumbar spine obtained in December 2025, demonstrating interval progression of metastatic disease with epidural involvement compared with prior imaging. (A) Sagittal T2-weighted lumbar spine image demonstrating a pathologic compression fracture of the L3 vertebral body with associated expansile soft-tissue mass extending into the epidural space, resulting in moderate to severe spinal canal stenosis and thecal sac compression. (B) Sagittal T2-weighted thoracic spine image demonstrating an epidural mass at the T6-T7 level within the posterior spinal canal, resulting in significant spinal canal narrowing consistent with metastatic epidural disease MRI: magnetic resonance imaging

In December 2025, the patient developed acute bilateral lower extremity weakness, sensory loss, and urinary retention. MRI of the cervical, thoracic, and lumbar spine obtained in December 2025 demonstrated marked interval disease progression compared with prior imaging in November 2025 (Figure 4). Findings included a new metastatic lesion in the C2 vertebral body, recurrent tumor within the prior T6-T7 laminectomy bed with epidural extension causing severe spinal canal stenosis, and new tumor progression at T11 with epidural encroachment and likely cord edema. Additional progression was noted throughout the lumbar spine, including an epidural tumor spanning L2-L4 and a pathologic compression fracture of L3, consistent with multifocal metastatic epidural disease.

Neurosurgical intervention was discussed; however, the patient elected to pursue palliative radiation therapy. He completed 10 fractions of radiation therapy to the thoracic and lumbar spine. Despite treatment, he remained paraplegic with a neurogenic bladder requiring chronic Foley catheterization.

His disease continued to progress with worsening systemic metastases, severe cancer-related pain requiring opioid escalation, and profound functional decline. Oncology determined that no further effective systemic treatment options were available. After goals-of-care discussions, he was transitioned to comfort-focused measures and died from progressive metastatic disease in February 2026.

## Discussion

This case highlights three clinically important themes: the aggressive natural history of primary mediastinal yolk sac tumors, the limited efficacy of PD-1 blockade in platinum-resistant germ cell malignancies, and the catastrophic neurologic consequences of malignant spinal cord compression during rapid metastatic progression.

PMNSGCTs are clinically and biologically high-risk tumors with outcomes inferior to gonadal germ cell tumors, particularly in nonseminomatous subtypes [1]. Large international analyses and subsequent reviews demonstrate that mediastinal primaries frequently fall into poor-risk categories and exhibit limited salvageability once platinum resistance develops [1,2]. In addition, emerging genomic data suggest that chemotherapy-resistant PMNSGCTs harbor biologic features associated with poor prognosis and therapeutic resistance, further distinguishing them from many gonadal counterparts [3]. This biologic backdrop helps contextualize the rapid recurrence observed in this patient despite intensive multimodal therapy, including cisplatin-based chemotherapy, surgical resection, and high-dose chemotherapy with autologous stem cell transplantation.

Importantly, the patient described in this report received guideline-concordant multimodal treatment, including cisplatin-based induction chemotherapy followed by surgical resection of residual disease, high-dose carboplatin and etoposide with autologous stem cell rescue, and subsequent gemcitabine-based salvage therapy prior to immunotherapy. Despite these aggressive and standard-of-care therapeutic approaches, durable disease control was not achieved, highlighting the particularly aggressive biology that can characterize mediastinal nonseminomatous germ cell tumors. Notably, minimal residual viable tumor following resection is generally considered a favorable prognostic factor in germ cell tumors [2], although this did not translate into durable disease control in the present case.

Serum AFP served as a consistent and clinically useful biomarker of disease activity throughout this patient’s course. In yolk sac tumors, AFP levels typically correlate with tumor burden, and rising values often precede or accompany radiographic progression. In this case, AFP repeatedly paralleled clinical deterioration across treatment lines. During pembrolizumab therapy, AFP increased from 44,053 to 90,123 ng/mL over approximately two months, corresponding to an estimated doubling time of less than 60 days. This rapid biochemical progression further supports aggressive tumor biology and is consistent with true disease progression rather than transient inflammatory change.

Checkpoint inhibition has shown limited efficacy in heavily pretreated germ cell tumors. In a phase 2 study of pembrolizumab in platinum-resistant germ cell tumors, objective responses were uncommon, and most patients experienced progressive disease as their best response [4]. Contemporary reviews similarly conclude that immune checkpoint inhibitors have not demonstrated reliable benefit in platinum-resistant germ cell tumor populations, particularly in aggressive nonseminomatous disease [5]. Combination checkpoint blockade has also produced low response rates and limited durability in heavily pretreated disease settings [6]. Taken together, these data align with the present case, in which pembrolizumab was followed by rapid biochemical progression and radiographic worsening with new metastatic sites. The tempo of progression and lack of clinical stabilization strongly support primary resistance to PD-1 inhibition. Notably, the rapid acceleration in tumor burden and the rise in AFP observed in this case may also be consistent with hyperprogressive disease, a phenomenon described in association with PD-1 inhibition.

Programmed death-ligand 1 (PD-L1) expression has been explored as a potential predictive biomarker for response to immune checkpoint inhibitors in several malignancies. However, PD-L1 staining was not performed on the initial tumor specimen in this case, and the predictive value of PD-L1 expression in germ cell tumors remains uncertain. Current evidence suggests that PD-L1 status alone may not reliably identify patients with germ cell tumors who will benefit from PD-1 blockade [5]. Moreover, even in malignancies where PD-L1 expression is routinely used, its predictive value remains imperfect, further underscoring the uncertainty of its role in germ cell tumors.

From a complications standpoint, this case underscores the devastating consequences of metastatic epidural disease. MRI remains the diagnostic modality of choice for malignant spinal cord compression due to its ability to define epidural tumor burden, degree of canal compromise, and associated cord edema [7,8]. In this patient, MRI demonstrated progressive multifocal spinal metastases with recurrent epidural disease at prior surgical sites and new high-grade stenosis at multiple levels, correlating with acute neurologic decline. Malignant spinal cord compression represents an oncologic emergency, and evidence-based management generally includes corticosteroids, urgent surgical decompression when appropriate, and radiation therapy [7,8]. However, neurologic recovery depends heavily on baseline function at presentation, and patients who present with complete motor deficits or prolonged compression often do not regain meaningful ambulation despite intervention [7]. Consistent with this, the patient remained paraplegic following palliative radiation and ultimately died from progressive metastatic disease.

Overall, this case illustrates that pembrolizumab should not be expected to provide reliable disease control in platinum-resistant PMNSGCT and reinforces the importance of close monitoring of tumor marker kinetics and neurologic symptoms in this high-risk population [1,4,7]. Continued investigation into novel therapeutic strategies remains urgently needed to improve outcomes for patients with aggressive mediastinal germ cell tumors [3,5,9].

## Conclusions

Primary mediastinal yolk sac tumors represent an aggressive subset of extragonadal germ cell malignancies with limited salvage options once platinum resistance develops. This case demonstrates rapid systemic progression despite multiple lines of therapy, including PD-1 inhibition with pembrolizumab, underscoring the lack of clinical benefit (primary resistance) to immune checkpoint blockade in heavily pretreated nonseminomatous germ cell tumors. Rising AFP levels correlated closely with radiographic progression and preceded catastrophic neurologic decline.

The subsequent development of multifocal spinal metastases and malignant spinal cord compression highlights the aggressive metastatic potential of platinum-resistant primary mediastinal disease and the importance of early recognition of neurologic symptoms in this population. Patients with known spinal metastases should be counseled on warning signs of cord compression, including back pain, weakness, sensory changes, and bladder dysfunction, to facilitate urgent evaluation. Clinicians should maintain realistic expectations regarding immunotherapy benefit in platinum-resistant germ cell tumors and monitor tumor marker kinetics closely to guide timely reassessment of treatment strategy. Novel therapeutic approaches are urgently needed to improve outcomes in this high-risk patient population.

## References

[1] Pini GM, Colecchia M (2022). Mediastinal germ cell tumors: a narrative review of their traits and aggressiveness features. Mediastinum.

[2] Bokemeyer C, Nichols CR, Droz JP (2002). Extragonadal germ cell tumors of the mediastinum and retroperitoneum: results from an international analysis. J Clin Oncol.

[3] Necchi A, Bratslavsky G, Chung J, Millis S, Gay LM, Ali SM, Ross JS (2019). Genomic features for therapeutic insights of chemotherapy-resistant, primary mediastinal nonseminomatous germ cell tumors and comparison with gonadal counterpart. Oncologist.

[4] Adra N, Einhorn LH, Althouse SK (2018). Phase II trial of pembrolizumab in patients with platinum refractory germ-cell tumors: a Hoosier Cancer Research Network Study GU14-206. Ann Oncol.

[5] Evmorfopoulos K, Marsitopoulos K, Karachalios R (2024). The immune landscape and immunotherapeutic strategies in platinum-refractory testicular germ cell tumors. Cancers (Basel).

[6] Klein O, Kee D, Markman B (2020). Immunotherapy of ipilimumab and nivolumab in patients with advanced neuroendocrine tumors: a subgroup analysis of the CA209-538 clinical trial for rare cancers. Clin Cancer Res.

[7] Loblaw DA, Mitera G, Ford M, Laperriere NJ (2012). A 2011 updated systematic review and clinical practice guideline for the management of malignant extradural spinal cord compression. Int J Radiat Oncol Biol Phys.

[8] Cole JS, Patchell RA (2008). Metastatic epidural spinal cord compression. Lancet Neurol.

[9] Zhang J, Chen Y, Liu L, Zhou M, Huang C, Guo C, Li S (2021). Clinicopathological features and prognosis of primary mediastinal malignant germ cell tumors: a retrospective single-institution analysis. Cancer Manag Res.

